# Evaluation of CME Arrival Prediction Using Ensemble Modeling Based on Heliospheric Imaging Observations

**DOI:** 10.1029/2020SW002553

**Published:** 2021-01-05

**Authors:** Tanja Amerstorfer, Jürgen Hinterreiter, Martin A. Reiss, Christian Möstl, Jackie A. Davies, Rachel L. Bailey, Andreas J. Weiss, Mateja Dumbović, Maike Bauer, Ute V. Amerstorfer, Richard A. Harrison

**Affiliations:** ^1^ Space Research Institute, Austrian Academy of Sciences Graz Austria; ^2^ Institute of Physics University of Graz Graz Austria; ^3^ Institute of Geodesy Graz University of Technology Graz Austria; ^4^ RAL Space, Rutherford Appleton Laboratory Didcot UK; ^5^ Conrad Observatory, Zentralanstalt für Meteorologie und Geodynamik Vienna Austria; ^6^ Hvar Observatory, Faculty of Geodesy University of Zagreb Zagreb Croatia

**Keywords:** space weather prediction, coronal mass ejections, ensemble modeling, heliospheric imaging

## Abstract

In this study, we evaluate a coronal mass ejection (CME) arrival prediction tool that utilizes the wide‐angle observations made by STEREO's heliospheric imagers (HI). The unsurpassable advantage of these imagers is the possibility to observe the evolution and propagation of a CME from close to the Sun out to 1 AU and beyond. We believe that by exploiting this capability, instead of relying on coronagraph observations only, it is possible to improve today's CME arrival time predictions. The ELlipse Evolution model based on HI observations (ELEvoHI) assumes that the CME frontal shape within the ecliptic plane is an ellipse and allows the CME to adjust to the ambient solar wind speed; that is, it is drag based. ELEvoHI is used to perform ensemble simulations by varying the CME frontal shape within given boundary conditions that are consistent with the observations made by HI. In this work, we evaluate different setups of the model by performing hindcasts for 15 well‐defined isolated CMEs that occurred when STEREO was near L4/5, between the end of 2008 and the beginning of 2011. In this way, we find a mean absolute error of between 6.2 ± 7.9 and 9.9 ± 13 hr depending on the model setup used. ELEvoHI is specified for using data from future space weather missions carrying HIs located at L5 or L1. It can also be used with near‐real‐time STEREO‐A HI beacon data to provide CME arrival predictions during the next ∼7 years when STEREO‐A is observing the Sun‐Earth space.

## Key Points

- CME prediction tool ELEvoHI is ready to be used in real time, based on STEREO‐A/HI beacon data
- Different model setups and inputs lead to large differences of the prediction accuracies
- Accurate modeling of the ambient solar wind is of particular importance to improve CME predictions

## 1 Introduction

As the main drivers of space weather events, coronal mass ejections (CMEs) are one of the most important subjects to be investigated as part of current solar‐terrestrial research. CMEs are impulsive outbursts of the solar corona, consisting of a magnetic flux rope that impounds coronal material and solar wind particles during its propagation through the interplanetary medium. Fast CMEs can reach speeds of up to 3,000 km s ^−1^ and, depending on their speeds and the characteristics of their intrinsic magnetic fields, can cause, for example, severe issues for satellites and disruptive geomagnetic disturbances at Earth (Farrugia et al., 2006; Gopalswamy et al., 2008; Huttunen et al., 2005; Tsurutani et al., 1988; Wilson, 1987). One of the most difficult CME properties to predict is the orientation of the magnetic field inside the CME, which is, at the same time, the most critical parameter due to the fact that a large southward magnetic field component facilitates the strongest geomagnetic storms. A large number of studies are currently tackling this task by developing new models that try to predict the orientation of the magnetic field at 1 AU (e.g., Kay et al., 2017; Kubicka et al., 2016; Möstl et al., 2018; Palmerio et al., 2017; Savani et al., 2015; Shiota & Kataoka, 2016; Singh et al., 2020; Verbeke et al., 2019).

Besides the magnetic field, the arrival speed of the CME plays an important role as high impact speeds, including those of the shock front driven by the CME, can intensify a geomagnetic disturbance (Gosling et al., 1991; Oliveira et al., 2018; Yue et al., 2010). Generally, geoeffectiveness is related to the dawn‐to‐dusk electric field and therefore to the flow speed (O'Brien & McPherron, 2000). While prediction of the orientation of the magnetic field within a CME is particularly difficult—especially due to the lack of magnetic field measurements in the corona—the prediction of the CME arrival time and speed can be carried out using different kinds of data and numerous prediction models. In particular, accurate prediction of the shock arrival time at Earth is crucial in order to be able to react accordingly to an expected disturbance. However, the timing and the probability of arrival at Earth are both still hard to predict. Wold et al. (2018) analyzed the real‐time predictions performed at the Community Coordinated Modeling Center (CCMC) using the WSA‐ENLIL+Cone model between the Years 2010 and 2016. They found the success ratio, reflecting the fraction of correct predictions, to be 0.4 and the false alarm ratio to be 0.6. This demonstrates the necessity of improving arrival time and probability prediction of CMEs.

Most prediction models rely on images from coronagraphs that observe the solar corona out to a maximum plane‐of‐sky distance of 30 *R*_⊙_ (e.g., Dumbović et al., 2018; Kay et al., 2020; Pluta et al., 2019; Singh et al., 2018). The big advantages of these observations are their availability in real time and their relatively simple interpretation. In coronagraph images, the inferred distances can be directly used without any consideration of Thomson scattering effects, which is commonly known as the plane‐of‐sky assumption. Additionally, the integration of the scattered photospheric light along the line of sight can be neglected, since the extent of a CME is rather small close to the Sun. The big drawback is the small field of view that corresponds to a maximum one seventh of the Sun‐Earth distance. Riley et al. (2018) analyzed the accuracy of models contributing to the CME scoreboard (https://kauai.ccmc.gsfc.nasa.gov/CMEscoreboard), a platform that is used by scientists and model developers to test their models in real time. It was found that the model with the best performance (WSA‐ENLIL+Cone run at National Oceanic and Atmospheric Administration/Space Weather Prediction Center [NOAA/SWPC]) achieved a mean absolute arrival time error of 13 hr with a standard deviation of ±15 h. The predictions evaluated were made in real time over a time range of almost 6 years; that is, the numbers in that study reflect the state of the art better than any of the other studies that covered only a small number of selected events.

Other instruments that enable CMEs to be observed in white light are the heliospheric imagers (HI;  Eyles et al., 2009) on board the Solar TErrestrial RElations Observatory (STEREO; Kaiser et al., 2008). These wide‐angle cameras image the space between the Sun and 1 AU and beyond. Due to their large field of view, line‐of‐sight integration is an important factor when interpreting these images and the plane‐of‐sky assumption is not valid for HI. Therefore, it is necessary to assume a certain longitudinal extent of the CME frontal shape, as well as being aware that it is not possible to follow the same part of the CME front during its propagation through the entire field of view of HI. One of the drawbacks of STEREO/HI data is that the near‐real‐time beacon data suffer from low time and spatial resolution and from data gaps; that is, it is expected that real‐time predictions based on HI beacon data cannot achieve the same accuracy as predictions based on HI science data (Tucker‐Hood et al., 2015). Now that STEREO‐A is again observing the space between Sun and Earth from an optimal vantage point, predictions using HI beacon data will no doubt be contributed to the CME scoreboard in the future. European Space Agency (ESA) is currently planning a space weather mission to the observationally advantageous Lagrange Point 5 (L5) of the Sun‐Earth system, located around 60° behind the Sun‐Earth line (Gibney, 2017). This mission is dedicated to space weather prediction and will, if funded, carry HI cameras providing real‐time data with quality comparable to STEREO/HI science data. This could be an important step forward to improving CME arrival time and speed prediction.

With regard to this and other possible future space weather monitoring missions carrying HI, we present a detailed evaluation of different model parameters and inputs to the ELlipse Evolution model based on single spacecraft HI observations (ELEvoHI; Amerstorfer et al., 2018; Rollett et al., 2016). ELEvoHI is designed to be operational in real time as soon as HI real‐time data are available with sufficient quality to be used by this model. We have found that small changes within the model, its parameters or inputs, can lead to a large difference in the CME arrival prediction. In the following sections, we investigate different ways of using ELEvoHI together with HI science data and compare these approaches to each other in order to identify the optimal model setup leading to the smallest prediction errors in time and speed.

## 2 Data

We use a list of 15 well‐observed (remotely and in situ) noninteracting Earth‐directed CMEs within the time range extending from the end of 2008 until the beginning of 2011 (Table 1). During this time, STEREO was in an ideal location (between 45° and 90° east and west of Earth) to observe Earth‐directed events. Unfortunately, due to low solar activity during these years, the number of fast CMEs in this interval is very small; that is, only one event arrived at Earth with a speed of more than 700 km s ^−1^ , while most of the events in the list were detected in situ with a speed of less than 400 km s ^−1^ .

**Table 1:** Overview of Events Used in this Study

No.	First observed by HI1 (UT)	HI observer	HEE longitude (°)	Arrival time (UT)	Arrival speed (km s ^−1^ )
1	2008‐12‐12 15:29	A	42.3	2008‐12‐17 03:35	355
2	2009‐01‐30 20:09	B	−46.1	2009‐02‐03 19:11	360
3	2009‐09‐03 23:29	A	59.6	2009‐09‐10 10:19	306
4	2010‐02‐03 14:49	A	64.7	2010‐02‐07 18:04	406
5	2010‐02‐03 20:49	B	−70.7	2010‐02‐07 18:04	406
6	2010‐03‐19 20:09	B	−71.4	2010‐03‐23 22:33	292
7	2010‐04‐03 12:09	A	67.5	2010‐04‐05 07:55	734
8	2010‐04‐08 06:49	A	67.8	2010‐04‐11 12:28	432
9	2010‐05‐23 22:09	A	71.6	2010‐05‐28 02:23	370
10	2010‐05‐24 00:09	B	−70.0	2010‐05‐28 02:23	370
11	2010‐06‐16 23:29	B	−69.8	2010‐06‐21 03:35	401
12	2010‐10‐26 16:10	B	−80.6	2010‐10‐31 02:09	366
13	2010‐12‐15 21:29	A	85.2	2010‐12‐19 20:23	381
14	2011‐01‐30 20:09	A	86.2	2011‐02‐04 01:55	376
15	2011‐01‐30 18:49	B	−93.0	2011‐02‐04 01:55	376

*Note*. The columns state the event number, the time and date when the CME was first visible in HI1, the observing STEREO spacecraft, the HEE longitude (i.e., the separation of the observing spacecraft from Earth), the in situ arrival time, and speed detected at Earth. This information was taken from the HELCATS project website. Dates are formatted as YYYY‐MM‐DD.

Parts of this study use coronagraph images provided by (1) the SOHO mission, with LASCO C2 and C3 (Brueckner et al., 1995), which observe the space around the Sun between 2 and 30 *R*_⊙_ in the plane of sky, and by (2) STEREO from two different vantage points, with COR2 (Howard et al., 2008) having a field of view extending from 2 to 15 *R*_⊙_ . For parts of this study, we use coronagraph observations from all three vantage points together to get an estimate of the CME shape. The most important data source for this study and the ELEvoHI model are provided by the HI on board STEREO. The HI instrument on each spacecraft consists of two white‐light wide‐angle cameras: HI1 having an angular field of view in the ecliptic of 4–24° from Sun center and HI2 having an angular field of view, again in the ecliptic, of 18–88°, roughly corresponding to a heliocentric distance of 1 AU. For this study, we used HI science data, having a time cadence of 40 min (HI1) and 2 hr (HI2). Three events in the list (Nos. 4 and 5, 9 and 10, and 14 and 15) are observed from STEREO‐A and STEREO‐B. CMEs viewed from the two different spacecraft are treated separately, that is, are not combined into a single prediction.

In order to evaluate the prediction accuracy of ELEvoHI, arrival times and speeds given in the ICMECAT (Möstl et al., 2017) catalog provided by the “Heliospheric Cataloguing, Analysis and Techniques Service“ (HELCATS) project (www.helcats‐fp7.eu) were used. This catalog lists, among those for other spacecraft, the interplanetary CME (ICME) shock arrivals detected by the Wind spacecraft (Lepping et al., 1995; Ogilvie et al., 1995) located at L1. Parts of this study rely on information about the solar wind speed at 1 AU detected by the Wind spacecraft, which is used as approximation for the ambient solar wind speed influencing the CME during its propagation (Section 4.2).

## 3 ELEvoHI at a Glance

The ELlipse Evolution model based on Heliospheric Imager data (ELEvoHI) was first presented by Rollett et al. (2016) as a single‐run model, where it was shown that including solar wind drag leads to an improvement of CME arrival time and speed predictions over the common HI prediction methods, such as fixed‐phi fitting (FPF) (Kahler & Webb, 2007; Rouillard et al., 2008), harmonic mean (Howard & Tappin, 2009; Lugaz et al., 2009), or self‐similar expansion fitting (SSEF) (Davies et al., 2012; Möstl & Davies, 2013). Allowing the CME to adjust its kinematics to the ambient solar wind flow particularly improves the arrival speed predictions, which has direct relevance to accurately predicting geomagnetic storm strength (Rollett et al., 2016).

Amerstorfer et al. (2018) introduced the ELEvoHI ensemble approach and tested it using a case study, in which a CME was detected in situ by two radially aligned spacecraft at 0.48 and 1.08 AU. The authors showed that it is possible to predict CME arrival at the observing spacecraft itself; that is, it is possible to predict a halo CME, supporting the idea of having an HI instrument positioned at L1.

**Figure 1:**
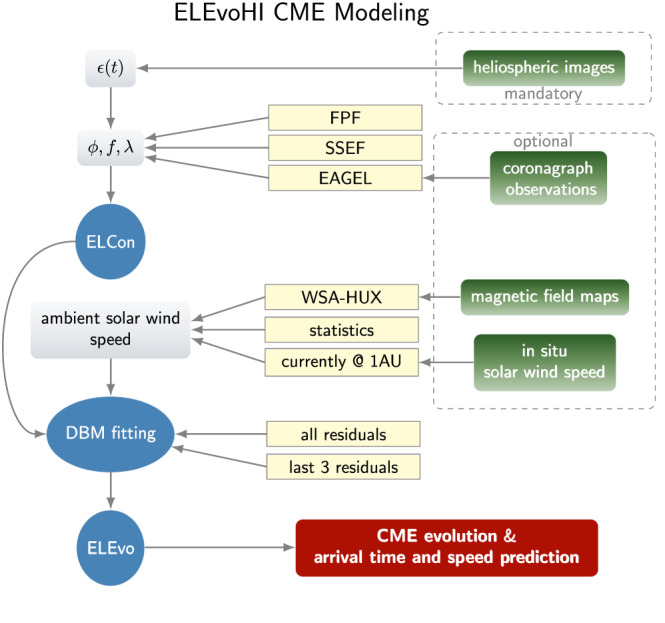
Schematic illustration of all parts contributing to an ELEvoHI ensemble prediction. The green boxes are the possible data used, and the blue boxes are the three main modules building ELEvoHI. The gray boxes are all input parameters needed. The yellow boxes show the possible sources of these input parameters grouped into three different parts that are tested in this study. The red box is the model output, that is, kinematical profiles and arrival time and speed predictions at the target of interest.

ELEvoHI is a combination of three main modules that derive parameters from observations to serve as input to the next module. Figure 1 presents the prediction scheme based on ELEvoHI ensemble modeling used in this paper. The left column shows different inputs (gray boxes) to the three main modules of ELEvoHI (blue ellipses), resulting in the modeling and prediction results (red box). The green boxes on the right show the different data that can be used to drive the model, while only data from HI are mandatory and all other data are optional. The middle part of the figure (yellow boxes) presents the three groups of inputs that this study investigates in order to identify their best combination (in terms of CME geometry, ambient solar wind speed, and drag‐based model [DBM] fitting). In the following paragraphs, the individual steps within ELEvoHI (blue circles in Figure 1) in its ensemble approach are briefly described:

The starting point is the CME time‐elongation track, *ϵ*(*t*), acquired from HI observations, usually from a time‐elongation map at fixed position angle. This track is converted from angular units to units of radial distance by ELEvoHI's built‐in procedure ELlipse Conversion (ELCon), based on an ensemble of assumed front shapes and propagation directions (see below). Detailed information on the ELCon conversion method can be found in Rollett et al. (2016).

In the next step, each ensemble member time‐distance track for the CME is fitted using an equation of motion based on the DBM given in (Vršnak et al., 2013)

(1) $$R(t)=\pm 1/\gamma \ln[1\pm \gamma (v_{\text{init}}-w)t]+wt+r_{\text{init}},$$

where *r*_init_ is the initial distance, *v*_init_ the initial speed and w is the ambient solar wind speed. The sign ± is positive when the CME is accelerating ( *v*_init_ < *w* ) and negative when it is decelerating ( *v*_init_ > *w* ) due to the drag force exerted by the ambient solar wind. The drag parameter, $\gamma =C_{D}\frac{A_{\text{CME}}\rho_{\text{sw}}}{m_{\text{CME}}}$, is the parameter that results from least squares fitting of the time‐distance track within the DBM fitting routine implemented in ELEvoHI. *C*_D_ is the drag coefficient assumed to equal 1, *A*_CME_ is the CME cross section that the drag is acting on, *m*_CME_ is the CME mass, and *ρ*_sw_ is the solar wind density. Within the DBM fitting procedure, *t*_init_ , the initial time of the fit, is defined manually by the user once for each event. Subsequently, *r*_init_ and *v*_init_ are derived separately for each ensemble member from the output of ELCon.

**Figure 2:**
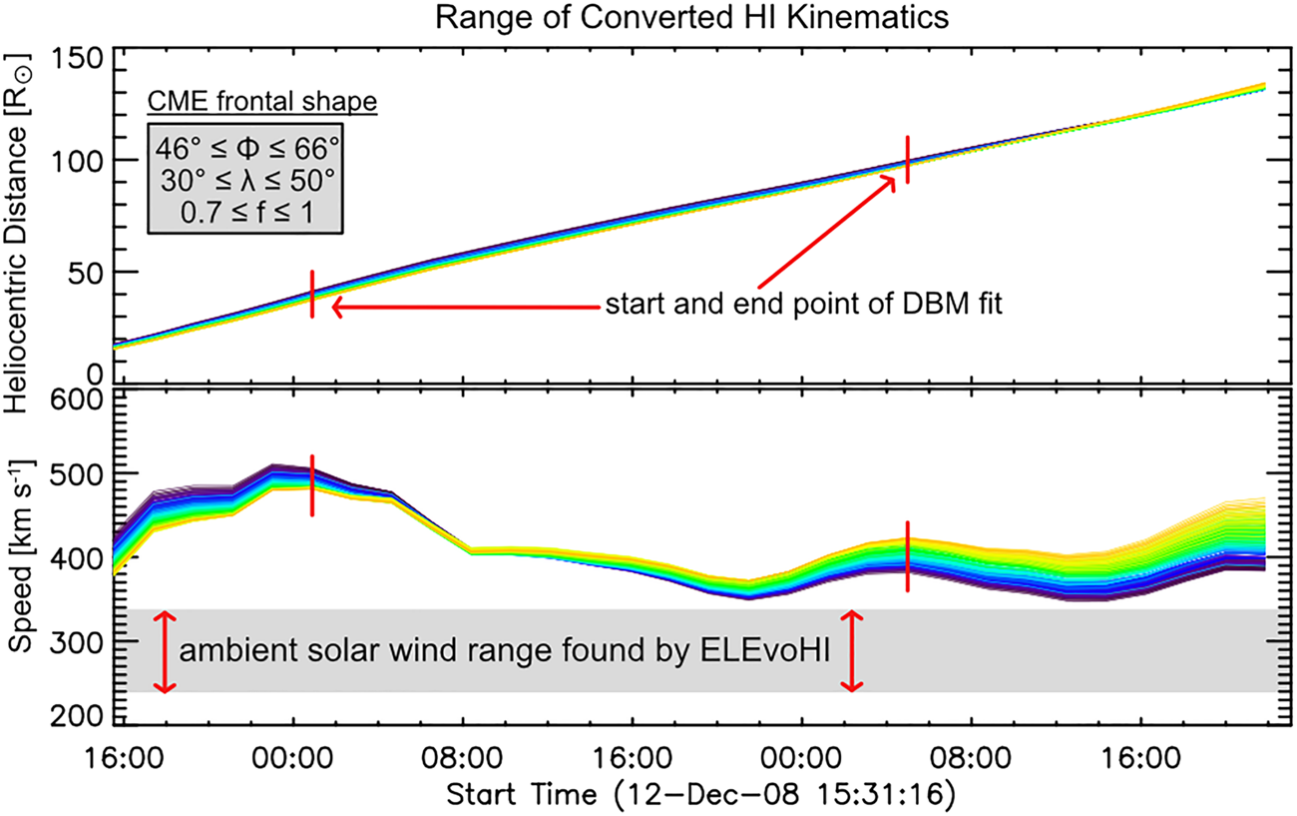
Range of HI kinematics (upper panel: time‐distance profiles, lower panel: time‐speed profiles) resulting from the input parameters corresponding to different CME frontal shapes and directions. The red vertical lines mark the start and end times of the HI data used for CME arrival prediction, and the gray shaded area in the lower panel illustrates the range of the ambient solar wind speed resulting from drag‐based fitting to the HI kinematics.

The procedure of defining *w* is described in section 4.2. Figure 2 demonstrates the approach of ELCon and the following DBM fitting for one example CME (CME No. 1 in Table 1). The upper panel shows the time‐distance profile derived from the STEREO‐A HI time‐elongation track by using 220 different combinations of frontal shape‐related input parameters (angular half width, *λ* , and inverse ellipse aspect ratio, *f*) and propagation direction, *ϕ* . Each of these three parameters is varied within a certain range to build an ensemble of different CME shapes, from each of which a prediction is made. Depending on the assumed angular width, aspect ratio and direction of the tracked feature, the derived kinematics differ for each ensemble member. The lower panel shows the interplanetary speed profiles of the CME apex derived by ELCon from each of the time‐distance profiles. The red vertical lines mark the start and the end point of the HI data used for DBM fitting (fits are not shown) and for making the CME arrival prediction.

The parameters obtained by DBM fitting serve as input for the ELlipse Evolution model (ELEvo; Möstl et al., 2015) that produces the arrival prediction. ELEvo runs the DBM by propagating the previously defined elliptical CME frontal shape in the also previously defined direction, which is different for each ensemble member, and predicts its arrival at any target of interest based on the drag parameter and ambient solar wind speed derived from DBM fitting.

In the following, we describe the different methods used to derive input parameters for ELEvoHI, such as information on the CME frontal shape, propagation direction, and the ambient solar wind speed. All of them are optional and can be replaced by a basic statistical estimation or a simple assumption.

## 4 Deriving the Input Parameters for ELEvoHI

### 4.1 Direction, Angular Width, and Curvature of the CME Front

Besides the time‐elongation track measured from HI observations, ELEvoHI needs information on the frontal shape, that is, *f* and *λ* , and the direction of motion of the CME. The latter can either be gained from HI observations or from coronagraph observations, which additionally provide the possibility to estimate the angular width.

#### 4.1.1 Ecliptic Cut Angles From GCS for ELEvoHI

The first potential method to provide *ϕ* and *λ* parameters used by ELEvoHI is based on the graduated cylindrical shell (GCS) fitting method (Thernisien, 2011; Thernisien et al., 2006, 2009). GCS fitting (implemented within SolarSoft, rtsccguicloud.pro) enables the manual fitting of a croissant‐shaped CME body to simultaneous images from coronagraphs observing from different vantage points. In our study, we use images from STEREO/COR2 from both sides, as well as LASCO/C2 and/or C3 images. Several shape‐related CME parameters can be adjusted within a widget tool until the best match with the CME visible within the coronagraph images is achieved. For our purposes, GCS is run as a part of the so‐called EAGEL (Ecliptic cut Angles from GCS for ELEvoHI) tool, which is described below.

**Figure 3:**
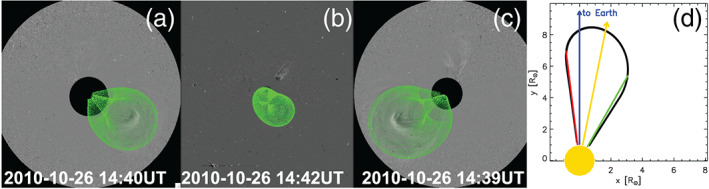
Example of a CME (Event No. 12) having a large inclination relative to the ecliptic plane. Panels (a)–(c) show the GCS reconstruction of the CME shape overlaid on STEREO‐A/COR2, SOHO/C3, and STEREO‐B/COR2 difference images. Panel (d) shows the ecliptic cut resulting from the EAGEL tool, with the blue arrow pointing toward Earth, the green and red lines defining the outer edges of the CME, and the yellow arrow pointing along the direction of motion used as input to ELEvoHI.

Within EAGEL the download and preprocessing of the coronagraph data is included in such a way that a CME is clearly recognizable in the images. Based on these images, GCS fitting of a CME is performed. EAGEL then creates an ecliptic cut of the wire frame of the fitted CME and calculates *λ* and *ϕ* with respect to Earth, STEREO‐A and STEREO‐B. ELEvoHI is operated in an ensemble mode, in which the input values of shape and direction are varied within a predefined range. In the case that inputs from EAGEL are used, *λ* and *ϕ* are each varied within ±10°. This range is chosen based on a previous study by Mierla et al. (2010), who cite this as the error range of these parameters when different observers manually fit the same CME using GCS. Figures 3a–3c show a GCS fit to one of the CMEs under study (No. 12 in Table 1). In case of this CME and due to the high tilt angle (≈−28°), the ecliptic cut conducted by the EAGEL tool corresponds to a very narrow structure as shown in Figure 3d). Because of the ±10° in *λ* and *ϕ* used in the ELEvoHI ensemble mode, the whole ensemble appears to be relatively wide compared to the input ecliptic cut. To build the ensemble, these inputs are varied using a step size of Δϕ=2° and Δλ=5°. The parameter *f*, which is related to the curvature of the front, is not obtained from the ecliptic cut but is, instead, varied between 0.7 (flat elliptical frontal shape) and 1 (circular frontal shape).

#### 4.1.2 FPF and SSEF Methods

In the study predicting CME arrival times and speeds using ELEvoHI performed by Rollett et al. (2016), the propagation direction was obtained from FPF and the same angular half‐width, namely, 35°, was used for every CME in the list. Although this is a quick and easy approach with no additional need for coronagraph data, it does not provide information about the true angular half‐width of the CME. With such information as input, we might be able to improve ELEvoHI's prediction accuracy. In the study by Amerstorfer et al. (2018), the information on the CME frontal shape was taken from an intersection of the GCS shape with the ecliptic plane (as discussed in section 4.1.1). That case study resulted in a prediction with very high precision.

To test the effect of assuming a finite CME width, we use the direction of motion from FPF as well as from SSEF (Davies et al., 2012; Möstl & Davies, 2013). These methods are analogous except that, in the latter, the CME is not assumed to be a point and one has to assume an angular half‐width for the circular shaped CME front. FPF and SSEF both perform a numerical fit to the time‐elongation profile of the CME track measured from HI observations; hence, they are based on the same input as ELEvoHI. Both methods assume a constant propagation direction and, in contrast to ELEvoHI, a constant propagation speed. We derive the propagation direction using SSEF assuming a half‐width of 45°. The propagation directions from both HI fitting methods were then used together with a range of 30–50° (and a step size of 5°) for the angular half‐width within ELEvoHI.

**Figure 4:**
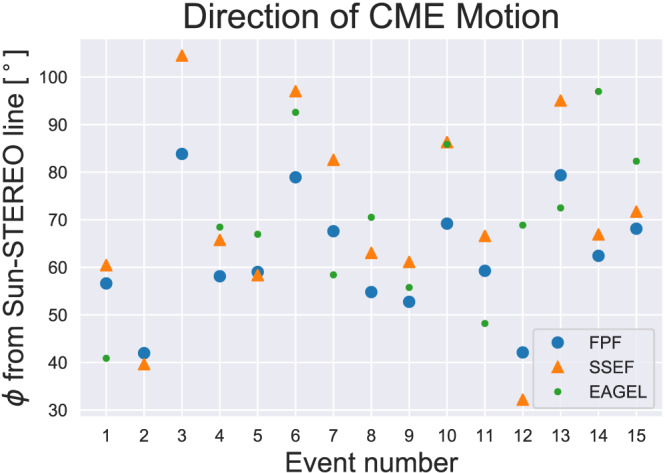
Absolute propagation directions relative to the Sun‐STEREO line derived from EAGEL (green), FPF (blue), and SSEF (orange). For two events GCS fitting was not possible. The mean absolute difference of the resulting directions is around 12.7°.

As a check, we compared the propagation directions resulting from FPF, SSEF, and EAGEL for the 15 CMEs under study and found that the mean absolute difference between the directions derived from the EAGEL approach and those from the HI fitting methods was around 14° and, between the two HI fitting methods, it was around 9°. Figure 4 shows the derived directions of motion derived using the three methods (EAGEL: green dot, FPF: blue circle, and SSEF: orange triangle) for each event studied. For Events 2 and 3, no GCS fit could be performed due to the faint nature of the CME structure within the coronagraph images. Therefore, for these events, we have no prediction based on model setups using information from the EAGEL method. It is expected that the direction of motion and the angular half‐width contribute significantly to the prediction accuracy. Amerstorfer et al. (2018) performed a sensitivity study that showed that, indeed, for the halo CME under study, the direction of motion had the biggest influence on the predicted transit time. However, this could be different for a side‐on view of a CME or for different events. It is important to emphasize that *λ* and *ϕ* are the only parameters in our model that dictate if Earth (or any other target) is hit by the CME or not.

For the ELEvoHI model setup test, as discussed in this section, we use the following inputs for the CME direction and angular half width:

(1) EAGEL direction and half‐width,
(2) FPF direction and predefined angular half‐width from 30–50°, and
(3) SSEF direction and predefined angular half‐width from 30–50°.

### 4.2 Ambient Solar Wind Speed

#### 4.2.1 In Situ Solar Wind Speed at 1 AU

In its current version, ELEvoHI accepts only a constant (in space and time) background solar wind input. Rollett et al. (2016) and Amerstorfer et al. (2018) assumed that the ambient solar wind at 1 AU is the same solar wind that influences the CME throughout its evolution; that is, the solar wind speed at 1 AU was used as input to ELEvoHI. Note that a background solar wind speed prescribed in this way is not truly representative of the actual background wind through which the CME propagates. In that approach, the minimum and maximum solar wind speed values over the time range of the HI data (either from STEREO‐A or STEREO‐B) are used for making the prediction and three values in between those minimum and maximum values, as the basis for the DBM fitting. Hence, five DBM fits are performed, and the optimal fit (defined below) gives us the background speed, which is further used to perform the prediction.

#### 4.2.2 Statistical Approach

In order to find a better method, we investigate whether the DBM fit is able to “decide” for itself which solar wind speed best fits the CME kinematics. To this end, we calculated the mean solar wind speed in OMNI data between the Years 2004 and 2018 to be 425 km s ^−1^ with a standard deviation of 100 km s ^−1^ . We use these values to define the speed range utilized for DBM fitting as the mean value ± twice the standard deviation. For each ensemble member, we perform 17 DBM fits corresponding to speeds from 225 to 625 km s ^−1^ in steps of 25 km s ^−1^ ; the optimal DBM fit then yields the background solar wind speed.

This approach allows the model to select from a wide range of possible background solar wind speeds for itself. This is possible because the HI kinematics are not compatible with every possible solar wind speed. Depending on the CME speed and its evolution, that is, whether the CME is decelerating, accelerating, or propagating with a constant speed, only some candidate solar wind speeds will result in a converging DBM fit. Due to the wide range of ensemble members, each having different kinematics (see gray area in the lower panel of Figure 2, the selected solar wind speed can be different for each ensemble member.

#### 4.2.3 Input From WSA‐HUX

As a third approach to deriving the background solar wind speed for input to ELEvoHI, we test the usage of the Wang‐Sheeley‐Arge and Heliospheric Upwind eXtrapolation models (WSA‐HUX). More specifically, we use magnetic maps of the photospheric field from the Global Oscillation Network Group (GONG) of the National Solar Observatory (NSO) as input to magnetic models of the solar corona. Using the Potential Field Source Surface (PFSS) model (Altschuler & Newkirk, 1969; Schatten et al., 1969) and the Schatten Current Sheet (SCS) model ( Schatten, 1971), we compute the global coronal magnetic field topology. While the PFSS model attempts to find the potential magnetic field solution in the corona with an outer boundary condition stating that the field is radial at the source surface at 2.5 *R*_⊙_ , the SCS model accounts for the latitudinal invariance of the radial magnetic field in the region between 2.5 and 5 *R*_⊙_ as observed in Ulysses field measurements (Wang & Sheeley, 1995). From the global magnetic field topology, we calculate the solar wind conditions near the Sun using the Wang‐Sheeley‐Arge model (WSA; Arge et al., 2003). To map the solar wind solutions from near Sun to Earth, we use the Heliospheric Upwind eXtrapolation model (HUX; Riley & Lionello, 2011), which simplifies the fluid momentum equation as much as possible. The HUX model solutions match the dynamic evolution predicted by global heliospheric magnetohydrodynamic (MHD) codes fairly well while having low processing power requirements. More details on the numerical framework can be found in Reiss et al. (2019).

**Figure 5:**
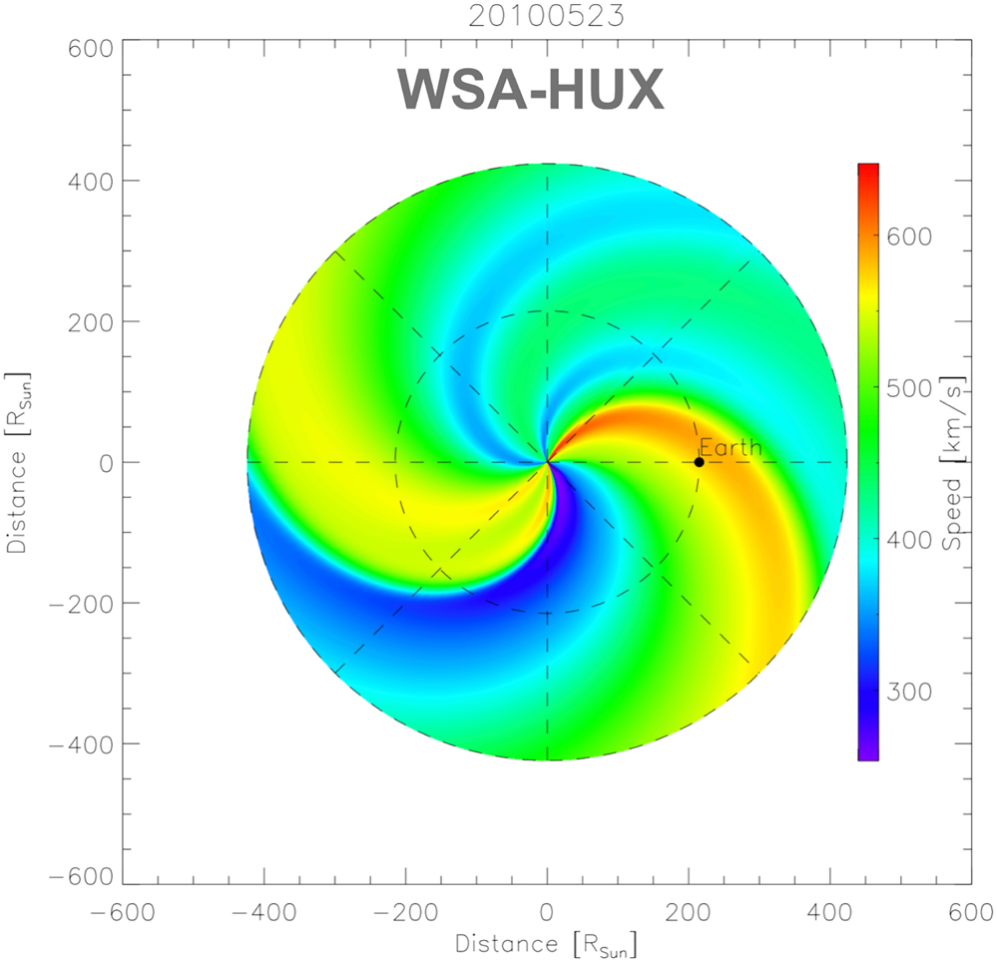
Example of the ambient solar wind speed for one event under study, observed by STEREO‐A and STEREO‐B (Events No. 9 and No. 10 in Table 1). The region of interest is extracted and averaged and serves as input to the ELEvoHI ensemble model.

Figure 5 presents the modeled ambient solar wind for one event under study (Events No. 9 and No. 10 in Table 1). For this method, we consider only that radial range of the heliosphere where the DBM fit is performed, that is, between the two red vertical lines indicated in Figure 2 (approximately 30–100 *R*_⊙_ ). In longitude, we use a range *ϕ* ± *λ* to define the area in which the solar wind is acting on a certain CME ensemble member. The median value of the solar wind speed within this sector is calculated and a range of ±100 km s ^−1^ is assumed. Over this range of ambient solar wind speeds, in steps of 25 km s ^−1^ , nine DBM fits are performed.

To test the ELEvoHI model setup, we use the three previously discussed methods to provide the source for the ambient solar wind speed, that is,

(1) in situ data from 1 AU,
(2) speed range derived from statistics, and
(3) modeled by WSA‐HUX model.

### 4.3 Definition of the “Optimal” DBM Fit

In the current version of ELEvoHI, the optimal DBM fit (out of several fits performed based on a range of input ambient solar wind speeds, as discussed in the previous section) is defined as the fit with the smallest mean residual to the time‐distance profile along the whole extent of the fitted curve. The ambient solar wind speed associated with the best DBM fit is then used for further modeling. Usually, the DBM fit is performed over a radial distance of around 30 to 100 *R*_⊙_ . Sometimes we find that the DBM best fit does not actually agree very well with the last fitted data points, which can have a significant influence on the prediction. Therefore, it is tested if using only the mean residual of the last three fitted points leads to a better prediction than considering the residuals of the whole fit. Note that, in both cases, the total number of data points that are fitted stays the same; that is, the track is fitted between the two end points that are manually chosen (vertical red lines in Figure 8). Only the evaluation of the residual differs in these two approaches.

For testing the ELEvoHI model setup, we use the two previously discussed methods for evaluating the DBM fit and choosing the most suitable background solar wind speed, that is,

(1) the smallest mean residual along the whole extent of the fit,
(2) the smallest mean residual of the last three fitted points.

### 4.4 Benchmark Model

In order to compare the results of the different ELEvoHI ensemble runs to a well‐established but simple prediction method that relies on HI data only, we use FPF (Kahler & Webb, 2007; Rouillard et al., 2008). The FPF method is the simplest of all such techniques based on HI data. It reduces the CME front to a point‐like feature and assumes a radial propagation direction at a constant propagation speed. The best fit equation of motion to the time‐elongation profile extracted from HI data provides an estimate of the arrival time and speed at the target of interest. We apply FPF to the same time‐elongation profiles as ELEvoHI and limit the track length to the start and end points between which the DBM fit is performed (red lines in Figure 2); that is, the same number of data points is used. Although the method is simple, its predictions are not significantly worse than the predictions from more sophisticated methods (Möstl et al., 2014). Using results from a benchmark model as a comparison provides the possibility to prove whether ELEvoHI is able to increase the prediction accuracy compared to the simpler FPF method.

## 5 Results

**Figure 6:**
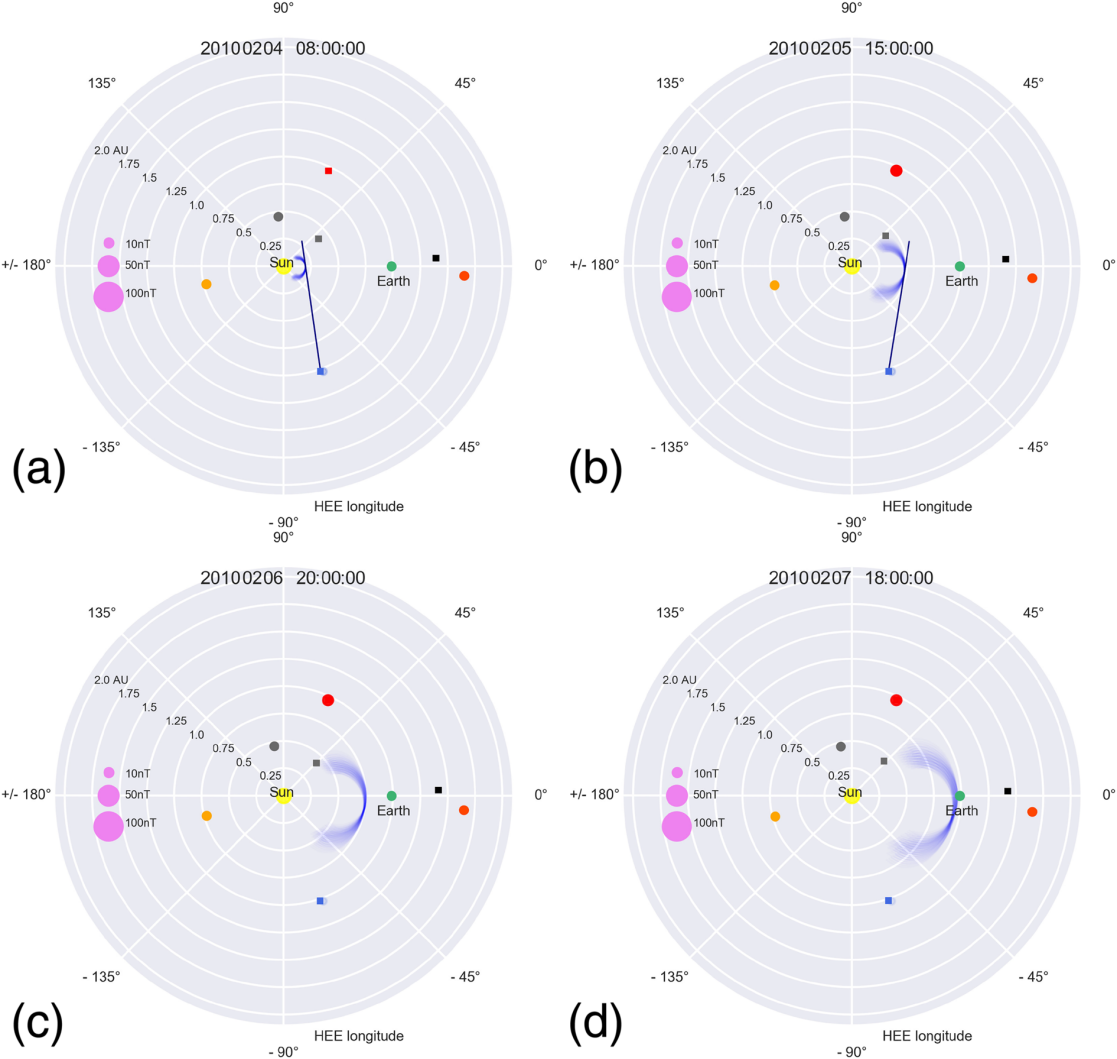
Four different time steps during ELEvoHI CME modeling for one example event (Event No. 5 in Table 2). Panel (a) shows the CME at the start of the DBM fit; the blue tangent corresponds to the measured HI‐elongation observed from STEREO‐B. Panel (b) shows the end of the DBM fit and the last HI‐elongation measurement used for the prediction. Panels (c) and (d) show additional times during CME evolution, while the latter corresponds to the time at which the CME was detected in situ at Earth. All movies and modeling results are available at figshare (https://doi.org/10.6084/m9.figshare.12333173.v1).

We perform 18 ensemble runs for each CME in our list of 15 events by combining three different ways of gaining the CME frontal shape/direction, three different approaches related to the ambient solar wind speed, and two different methods of defining the best DBM fit. Every ensemble run consists of 220 ensemble members (resulting from varying *λ* , *f*, and *ϕ* input parameters within certain ranges); that is, for each event, we perform 3,960 predictions with 59,400 predictions in total. We calculate the median, the mean, and the standard deviation of the distribution of predictions of the arrival time at Earth for each of the 18 ensembles and for each of the 15 CMEs. Figure 6 shows four different time steps of the ELEvoHI simulation result for one example event (No. 5 in Table 1, 2). Figures 6a and 6b correspond to the start and end times of the DBM fit; the blue tangent represents the corresponding HI elongation measurement. Figure 6c and 6d show later time steps of the prediction, for which no HI data were used (hence no blue tangent). Figure 6d presents the time of the in situ arrival detection.

**Table 2:** Accuracy of Each Model Setup, Sorted by the Mean Absolute Error (MAE)

MAE(*t*) (hr)	ME(*t*) (hr)	RMSE(*t*) (hr)	MSTD(*t*) (hr)	MAE(v) (km s ^−1^ )	ME(*v*) (km s ^−1^ )	RMSE(*v*) (km s ^−1^ )	MSTD(*v*) (km s ^−1^ )	Model setup
6.2	0.1	7.9	5.2	39.0	17.8	46.7	39.3	FPF_WSA‐HUX_all
6.4	−0.1	8.0	5.1	36.8	18.6	45.1	38.6	FPF_WSA‐HUX_last
7.3	1.5	9.2	5.6	49.3	23.6	56.6	40.5	SSEF_WSA‐HUX_all
7.3	−2.5	10.0	3.5	62.1	34.0	74.5	70.2	EAGEL_WSA‐HUX_all
7.4	1.3	9.1	5.6	47.7	22.2	55.3	40.7	SSEF_WSA‐HUX_last
7.5	−2.8	10.1	3.5	60.8	35.3	73.4	70.4	EAGEL_WSA‐HUX_last
7.7	−1.8	10.3	4.5	65.0	33.5	75.3	71.0	EAGEL_stats_all
7.8	−2.1	10.3	4.6	62.7	35.8	72.9	71.7	EAGEL_stats_last
8.0	−2.1	10.1	3.9	61.9	34.9	75.0	64.6	EAGEL_L1_all
8.0	−2.1	10.2	3.9	62.4	34.4	75.3	64.7	EAGEL_L1_last
8.6	2.7	12.2	6.6	45.5	11.2	55.8	43.9	FPF_stats_all
8.8	2.6	12.2	6.6	43.4	11.4	54.5	43.5	FPF_stats_last
8.9	3.3	11.8	6.5	51.7	17.2	62.9	42.8	SSEF_stats_all
9.1	3.2	11.8	6.5	49.6	15.4	61.1	44.5	SSEF_stats_last
9.1	2.4	13.1	6.7	51.0	15.7	63.9	45.8	FPF_L1_all
9.1	2.4	13.1	6.6	50.3	15.5	63.6	45.4	FPF_L1_last
9.8	2.7	13.0	6.5	61.0	25.6	80.4	45.7	SSEF_L1_all
9.9	2.6	13.0	6.5	61.2	25.4	80.4	46.2	SSEF_L1_last

*Note*. MAE, the mean error (ME), the root‐mean‐square error (RMSE), and the mean standard deviation of the arrival time (*t*) and speed (*v*) prediction are given. The last column lists the corresponding model setup indicating the inputs for direction (and shape in case of EAGEL), solar wind, and the way of defining the best DBM fit. The benchmark model FPF results for the arrival time prediction in an MAE of 7.8 hr, an ME of 2.6 hr, an MSTD of 10.5 hr, and a RMSE of 10.1 hr. For the arrival speed prediction FPF results in an MAE of *X* hr, an ME of *X* hr, an MSTD of *X* hr, and a RMSE of 60 hr.

Overall, ELEvoHI achieves a mean absolute error (MAE) of 8.2 ± 5.5 hr, a root‐mean‐square error (RMSE) of 11.1 hr, and a mean error (ME) of +0.8 hr, the latter indicating that our model neither overestimates nor underestimates the transit time. Here, it is important to emphasize that these values arose from analyzing a small set of 15 isolated (noninteracting) events that were not predicted in real time. Therefore, one has to be careful when comparing these results with the results of studies dealing with real‐time predictions. In the following paragraphs, we evaluate the performance of the different model setups.

Table 2 lists the MAE, the ME, the RMSE, and the mean standard deviation (MSTD) of the difference between predicted and observed arrival time, *t*, and speed, *v*, for each of the 18 different model setups. Negative values correspond to an underestimated transit time; hence, the event was predicted to arrive earlier than it actually did. In the case of the arrival speed prediction, negative values correspond to an underestimated arrival speed. The results are ordered from smallest to largest MAE in arrival time, revealing that the six setups using the WSA‐HUX output as input for the ambient solar wind estimate lead to the most accurate predictions. The benchmark FPF technique leads to an MAE of 7.8 hr with an MSTD of 10.5 hr and an ME of 2.6 hr, which means that FPF has a tendency to overestimate the transit time. Considering the underlying geometry assumed by FPF, this is not surprising. FPF reduces the CME front to a single point and assumes that this point is tracked throughout the CME's propagation. Being conscious of the fact that CMEs can be extremely large‐scale structures, it is clear that this is an oversimplification. Additionally, our CME sample almost exclusively consists of slow CMEs for which the assumption of constant propagation speed is usually close to reality. The faster the CME and hence the larger its likely deceleration, the larger the error due to a constant speed assumption (Lugaz et al., 2011). However, we cannot dismiss the result that FPF performs as well as ELEvoHI (when averaging over all model setups) for the chosen set of CMEs. Again, it can be shown that a more sophisticated method is no guarantee of a better prediction as already demonstrated by Vršnak et al. (2014), who compared the performance of the DBM and the WSA‐Enlil+Cone model based on a list of 50 CMEs. The authors found that the two methods predicted the CME arrival time with an MAE of 14.8 and 14.1 hr, respectively (for real‐time predictions). Fortunately, this does not mean that we have already reached the best possible prediction accuracy; improving a method can still reap rewards. As Table 2 shows, ELEvoHI based on phi from FPF can outperform the benchmark FPF when part of a more sophisticated model setup, for example, when coupled with WSA‐HUX as the source of the solar wind.

**Figure 7:**
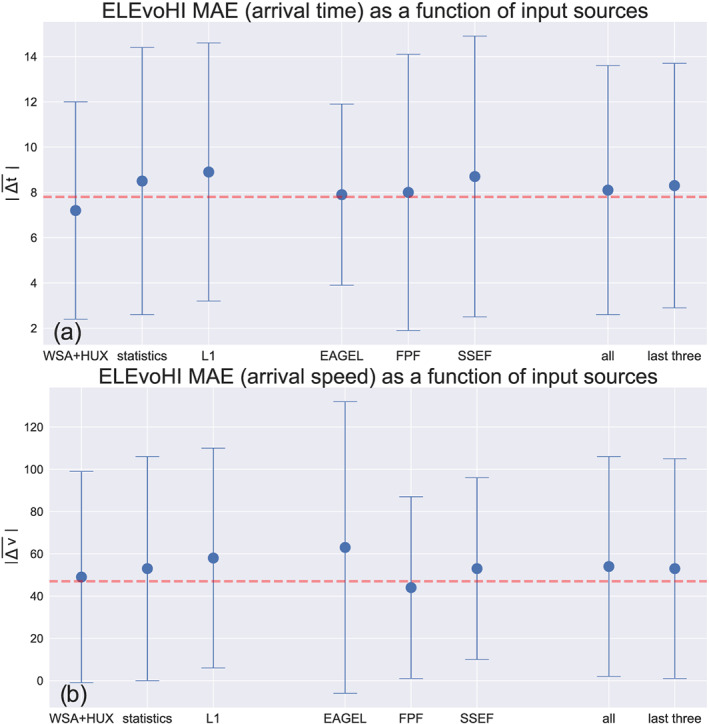
ELEvoHI MAE of (a) arrival time and (b) speed prediction corresponding to each source of input parameter. The left set of bars correspond to the three different sources of ambient solar wind input, the middle set of bars correspond to the three different sources of propagation direction (and CME frontal shape in case of EAGEL), and the right set of bars show the results that correspond to the two different ways of defining the best DBM fit. The error bars mark the standard deviation of the predictions. The horizontal dashed lines represent the MAE of the benchmark model, FPF.

Figure 7 shows the performance of the different model setups, grouped by the input type. The left/middle/right bars show the MAE and the MSTD of predictions based on different kinds of solar wind input/frontal shape/direction/best DBM fit. For all runs that use WSA‐HUX, we find an MAE of 7.2 ± 4.8 hr, using statistical background wind information results in an MAE of 8.5 ± 5.9 hr and using the in situ solar wind speed from L1 we find an MAE of 9.0 ± 5.7 hr. We find that using WSA‐HUX as the background solar wind source results in six events being predicted better (i.e., with the smallest MAE) than using other solar wind sources (statistical background wind: 5, L1: 4). For one event in the list, no DBM fit converged when using WSA‐HUX as input, and therefore, no prediction was possible. The reason for that is that the solar wind speed range derived from WSA‐HUX was not compatible with the HI kinematics. This can be the case when the HI data imply CME deceleration while the provided ambient solar wind speed is larger than the CME speed. In this situation, no physical solution relating to drag can be found as the CME cannot be decelerated below the speed of the ambient solar wind flow. This demonstrates the additional value of the whole approach because of the way that it avoids inappropriate values for the background solar wind speed. This is a distinct advantage over methods that only rely on coronagraph data and provide no possibility to validate the background solar wind speed used in the model.

Comparing the predictions based on different sources of CME frontal shape/direction input, we find that the input from the EAGEL tool leads to an MAE of 7.9 ± 4.0 hr and predictions based on FPF and SSEF result in an MAE of 8.0 ± 6.1 hr and 8.7 ± 6.2, respectively. Interestingly, predictions based on EAGEL result in a smaller MSTD (related to the arrival time) than those based on FPF or SSEF. This is the result of a smaller angular width derived by EAGEL for some events in the list than the value assumed for the predictions based on FPF and SSEF propagation direction. In terms of the number of best predictions, using EAGEL results in the most accurate arrival time predictions for six of the CMEs (SSEF: 5, FPF: 4). The last comparison is made between the results of the two methods for defining the optimal DBM fit. Here, we find no significant difference between the results of the method that takes into account the residuals of the whole fit (8.1 ± 5.5 hr) and the method that uses residuals of the last three fitted points only (8.2 ± 5.4 hr). Nevertheless, using the whole fit for evaluation leads to the best prediction for 9 out of the 15 CMEs and using only the last three residuals leads to the best prediction for the other six CMEs. Contrary to our conclusion made above, this means that using the residuals over the whole fit has a clear advantage over using only the last three residuals to judge the fit.

Evaluating ELEvoHI's ability to predict CME arrival speed based on each input parameter (Figure 7b), we find that using WSA‐HUX as background solar wind input, results in an MAE of 49 ± 50 km s ^−1^ (L1: 58 ± 52 km s ^−1^ , statistical background wind: 53 ± 53 km s ^−1^ ). Using input from EAGEL yields an MAE of 63 ± 69 km s ^−1^ (FPF: 44 ± 43 km s ^−1^ , SSEF: 53 ± 43 km s ^−1^ ). Judging the best DBM fit by the residuals of the whole fit gives 68 ± 52 km s ^−1^ (last three residuals: 66 ± 52 km s ^−1^ ). In case of the CME arrival speed prediction, the setup used appears to make little difference.

**Figure 8:**
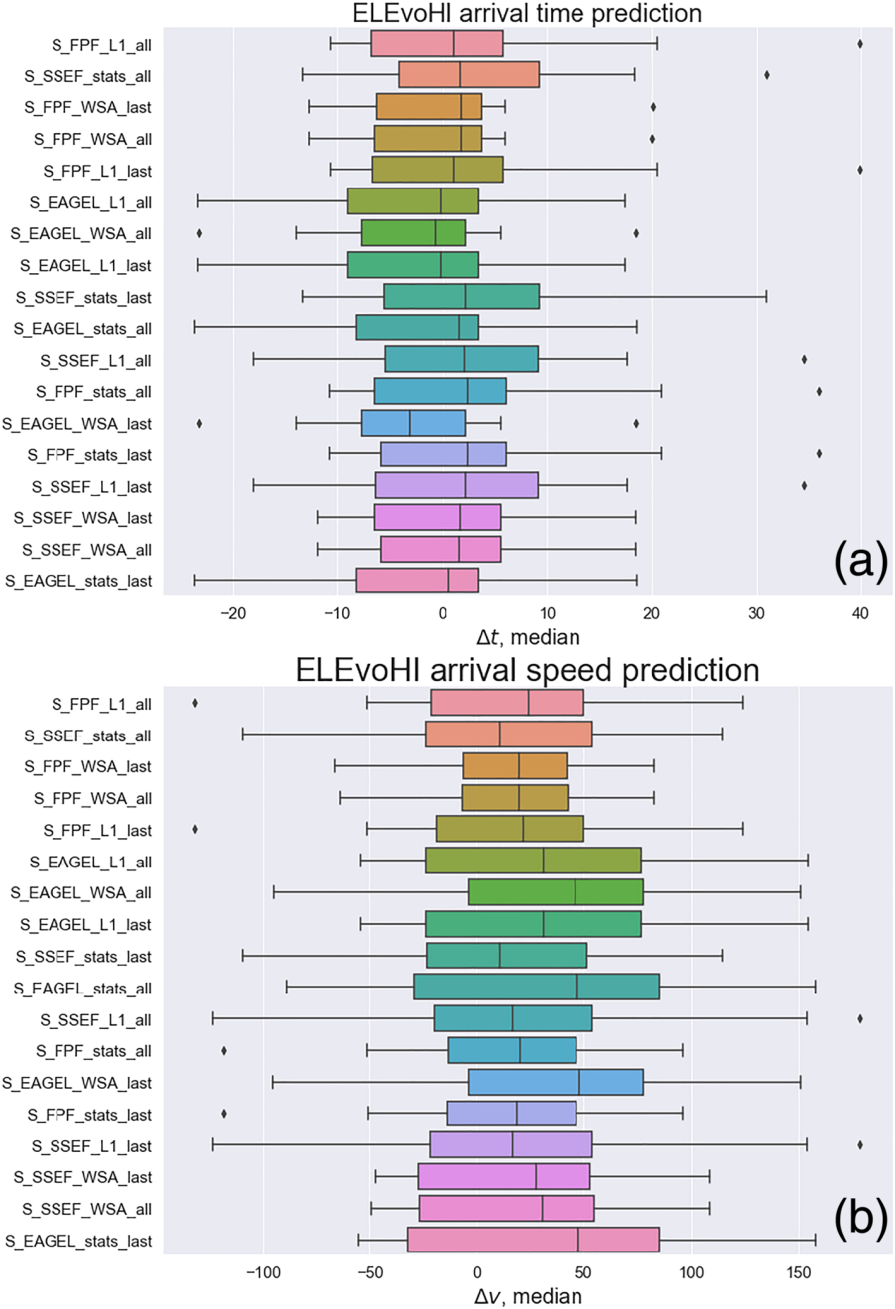
Overview of the prediction accuracy for every model setup tested. Panel (a) presents the prediction accuracy for the arrival time and panel (b) for the arrival speed. The vertical lines within the boxes correspond to the median values, the boxes are delimited by the first and the third quartile, and the whiskers correspond to 1.5 times the interquartile range; the diamonds represent outliers.

Figure 8a shows an overview of the performance of all of the different model setups as box and whiskers plots, based on the difference between predicted and actual arrival time for all events and all runs (∼3,000 runs per box). For almost every setup the median is quite close to 0. This shows us that ELEvoHI has no bias toward providing predictions that are either too early or too late. As noted above, the overall RMSE(*t*) is 11 hr and the MAE(*t*) is 8.2 ± 5.5 hr, reflecting the actual prediction accuracy. The overall RMSE(*v*) is 66 km s ^−1^ , and the MAE(*v*) is 53 km s ^−1^ . Figure 8b shows the analogous plot for arrival speed. Overall, ELEvoHI provides an MAE in the arrival speed prediction of 53 ± 51 km s ^−1^ , an RMSE of 66 km s ^−1^ , and ME of 23 km s ^−1^ . The latter means that ELEvoHI is not biased toward producing arrival speed predictions that are either too fast or too slow.

**Figure 9:**
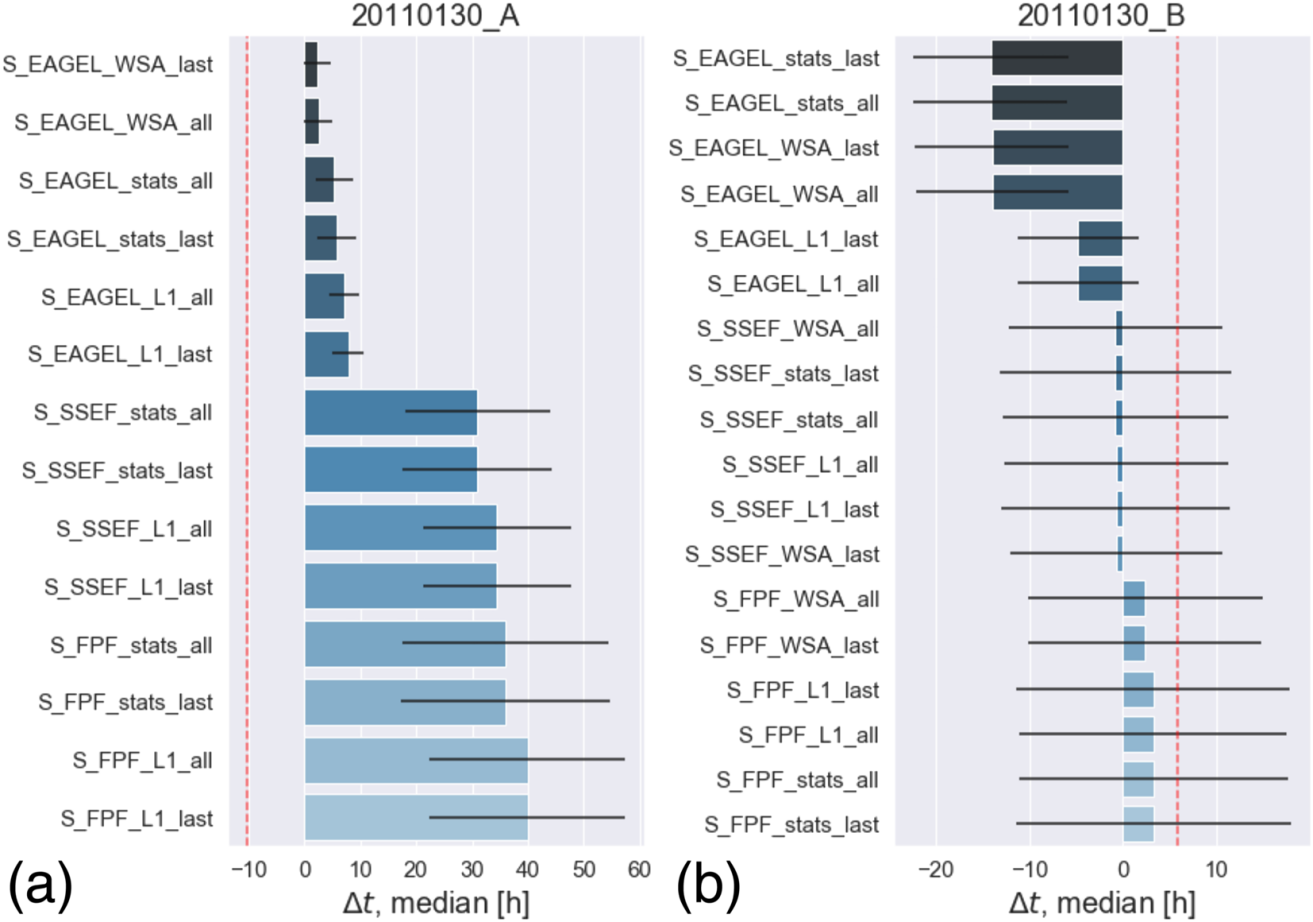
Comparison of ELEvoHI predictions for one example event (Nos. 14 and 15 in Table 1) performed separately for the two different vantage points, that is, from STEREO‐A and B, respectively. The dashed red line shows the result of the benchmark model.

Some of the events under study (Nos. 4 and 5, 9 and 10, and 14 and 15 in Table 1) are observed and predicted from two separate vantage points, namely, from STEREO‐A and STEREO‐B. Provided that the assumption of an elliptical self‐similarly expanding structure is true, we might suppose that the predictions of an event observed from different sides agree well. Figure 9 refutes this assumption by presenting the arrival time predictions for a CME launched on 30 January 2011. In Figure 9a the predictions based on STEREO‐A/HI data (No. 14 in Table 1) are shown, and Figure 9b presents the predictions based on STEREO‐B/HI data (No. 15 in Table 1). Interestingly, the results are highly dependent on the model setup used. For the view from STEREO‐A, EAGEL+WSA‐HUX input seems to be the best choice compared to the predictions based on the two HI fitting methods that lead to an error between 30 and 40 hr. The combination of SSEF/FPF and WSA‐HUX was not possible for this event from the vantage point of STEREO‐A because the ambient solar wind speed range provided by WSA‐HUX did not agree with the HI kinematics. Contrariwise, from the vantage point of STEREO‐B, the EAGEL+WSA‐HUX setup leads to an error of more than 10 hr, while the predictions based on input directions derived from SSEF almost exactly match the in situ arrival time. A more detailed analysis on CMEs observed stereoscopically and modeled by ELEvoHI will be presented in a study by Hinterreiter et al. (in preparation for Space Weather).

This comparison shows that the current assumptions within ELEvoHI, that is, constant ambient solar wind speed and elliptical CME frontal shape, are not correct for every event. When the CME is observed and predicted from the two different vantage points, the results can differ significantly; with the correct assumptions in place for a specific CME, this should not be the case. Therefore, including a deformable shape within ELEvoHI to simulate CME interaction with structures in the ambient solar wind might lead to an improvement of the predictions. Indeed, observations from more than one vantage point could be used to help constrain the shape and kinematics of the CME leading to such an improvement in the arrival prediction accuracies. This finding supports the benefit of having HI observations from two separate vantage points, for example, L1 and L5.

## 6 Applicability for Real‐Time Predictions

By far the fastest and according to our findings in this study, a relatively satisfying way to setup ELEvoHI, is using a combination of FPF and the statistical ambient solar wind approach. FPF uses the same data as needed by ELEvoHI, that is, the HI time‐elongation track. The FPF fitting method yields the propagation direction needed by ELEvoHI, while the half‐width within the ecliptic plane can be assumed to be between 30° and 50° (it can be assumed, indeed, to be any other value). The statistical solar wind approach is directly implemented within the ELEvoHI model. As shown above, this setup leads to an MAE in arrival time of 8.6 hr and an ME of 2.7 hr. However, if an ambient solar wind solution is available in real time (e.g., the WSA‐HUX or similar), ELEvoHI can achieve an MAE of 6.2 hr with an ME of 0.1 hr—still without the necessity for additional coronagraph data or the need for manual fitting to these images. Of course, we always need to keep in mind that these values are derived from a predefined set of very well observed, and isolated, events and from HI science quality data that is currently not available in real time. However, HI beacon data are available in near real time and can serve as input to ELEvoHI since STEREO‐A/HI is already close to L5 and is observing the space between the Sun and Earth—hopefully until 2027, when it will be around L4. An additional possibility for having HI real‐time data available in the future might be provided by the Polarimeter to Unify the Corona and Heliosphere (PUNCH) mission. PUNCH will be launched in 2023 and will operate in low Earth orbit.

For real‐time predictions, it is of the utmost importance to be able to include an estimate of the arrival probability with a CME prediction. Currently, ELEvoHI simply calculates this as the ratio of the number of ensemble members that are predicted to hit the target to the total ensemble size. This is going to be updated in the near future, to give predicted flank hits a lower weighting. In addition, we have noticed that for flank hits, the arrival time error tends to be larger than expected and the transit time is overestimated. This could be due to the elliptical shape of the front resulting in highly curved flanks. In the future, we will examine if we can find a suitable approach to deal with these strongly bent flanks to avoid such extreme delays when predicting a flank encounter.

## 7 Summary and Conclusions

In this work we studied 18 different combinations of inputs to run the HI‐based ensemble CME arrival prediction model, ELEvoHI, in order to ascertain the setup leading to the most accurate arrival time and speed predictions. As input for the ambient solar wind that influences the drag‐based propagation of the modeled CME we used (1) the WSA‐HUX background solar wind model, (2) an approach of simply providing a range of possible solar wind speeds (225–625 km s ^−1^ ) derived from 14 years of observations at L1, and (3) the solar wind speed measured in situ at L1 during the evolution of the CME. We found that having a more accurate ambient solar wind as input leads to significantly better arrival time prediction. Using input from WSA‐HUX improves the MAE by an hour, compared to simply providing a range for solar wind speeds, and leads to almost 2 hr improvement on MAE over the usage of L1 solar wind speed.

To analyze the influence of the CME frontal shape/propagation direction on ELEvoHI predictions, we compared three different sources of *λ* and *ϕ* : (1) Coronagraph images were used to perform a GCS fit to derive the 3‐D shape of the CME. The intersection of this 3‐D front with the ecliptic plane provides a 2‐D structure from which the measured angular half‐width and direction were input to ELEvoHI; (2) the FPF; and (3) the SSEF HI fitting methods, which only provide the direction of motion. In these cases, we had to assume a half‐width (we chose a range between 30° and 50°). In all cases we had to assume *f* to vary between 0.7 and 1, while a value of 1 corresponds to a circular frontal shape. Surprisingly, approach 1 did not lead to a significantly more accurate prediction than using FPF or SSEF and simply assuming the half‐width to lie within a certain range. One possible reason for this might be different direction of motion within the coronagraph field of view compared to that within the HI field of view. Another reason might be an ongoing rotation of the CME, leading to a change in its angular half‐width within the ecliptic. However, this is a surprising result as one would expect a more data‐oriented input (regarding the CME shape) to lead to better predictions.

The third aspect of the model setup that we tested is the model‐intrinsic procedure to determine the optimal DBM fit to the HI kinematics. This process defines the ambient solar wind speed, which is further used as a basis for the arrival prediction. Several DBM fits were performed over the provided range of solar wind speeds; the best DBM fit then determined the optimal ambient solar wind speed and drag parameter. We compared two ways of defining the best DBM fit, namely, the fit with the minimum value of the mean residual of (1) the whole fit or (2) the last three points of the fit. We found that both procedures lead to similar results, but with using the residuals of the whole fit leading to slightly better predictions.

Based on this study, we are now able to operate ELEvoHI to gain the best possible arrival predictions. Our results emphasize the importance of an accurate ambient solar wind model, as the solar wind heavily influences the drag‐based evolution of the CME. In the future, an interesting advancement might be to include a range of values for the solar wind to contribute to the ensemble instead of deriving only a single value per shape/direction setup. Another logical next step would be to release ELEvoHI from its rigid elliptical shape and to allow deformation due to the influence of the ambient solar wind. In any case, with ELEvoHI, we are prepared for real‐time CME arrival predictions, once a new HI observer is delivering high‐quality data in real time.

## Data Availability

Data are from the following sites: STEREO/HI (https://www.ukssdc.ac.uk/solar/stereo/data.html), STEREO/COR2 and SoHO/LASCO (https://sdac.virtualsolar.org/cgi/search), and NSO/GONG (https://gong.nso.edu/data/magmap/). ELEvoHI is available on GitHub (under https://github.com/tamerstorfer/ELEvoHI/releases/tag/v1.0.0.0). The visualization of each prediction result, that is, movies and figures, can be downloaded from figshare (https://doi.org/10.6084/m9.figshare.12333173.v1).
